# IHR-PVS National Bridging Workshop for Somalia: An interactive and participatory approach for operationalizing the One Health roadmap

**DOI:** 10.1016/j.onehlt.2024.100858

**Published:** 2024-07-14

**Authors:** Abdinasir Yusuf Osman, Asma Saidouni, Lillian Wayua Wambua, Heba Mahrous, Sk Md Mamunur Rahman Malik, Mutaawe Lubogo, Reinhilde Van de Weerdt, Ali Hadji Adam, Hassan Hussien Mohamed, Khadija Al Makhzoumi, Guled Abdijalil Ali, Mohamed Omar Nur, Sonia Fevre, Gerald Mucheru, Sophycate Njue, Alain Okito Mosindo, Kaitlin Sandhaus, Rosita Claesson Wigand, Claire Standley, Erin Sorrell, Richard Kock, Javier Guitian, Alimuddin Zumla, Osman Dar, Siobhan M. Mor

**Affiliations:** a World Organization for Animal Health (WOAH) Collaborating Centre in Risk Analysis and Modelling, Food and Agriculture Organization of the United Nations (FAO) Reference Centre for Veterinary Epidemiology, Veterinary Epidemiology, Economics and Public Health, Department of Pathobiology and Population Sciences, Royal Veterinary College, London, UK; b Ministry of Health, Mogadishu, Somalia; c World Health Organization, Regional Office for the Eastern Mediterranean, Cairo, Egypt; d World Organisation for Animal Health Sub-Regional Representation for Eastern Africa, Nairobi, Kenya; e World Health Organization, Country Office, Mogadishu, Somalia; f Ministry of Livestock Forestry and Range, Mogadishu, Somalia; g Ministry of Environment and Climate Change, Mogadishu, Somalia; h Food and Agriculture Organization of the United Nations, Headquarters, Rome, Italy; i Food and Agriculture Organization of the United Nations, Country Office, Mogadishu, Somalia; j United Nations Environment Programme, Africa Office, Nairobi, Kenya; k Global Implementation Solutions, Kisumu, Kenya; l Public Health Agency of Sweden, Solna, Sweden; m Center for Global Health Science and Security, Georgetown University, Washington, DC, USA; n Center for Health Security, Johns Hopkins, Bloomberg School of Public Health, Baltimore, MD, USA; o National Institute for Health and Care Research Biomedical Research Centre, University College London Hospitals NHS Foundation Trust, London, UK; p Department of Infection, Division of Infection and Immunity, University College London, London, UK; q Global Health Programme, Royal Institute of International Affairs, London, UK; r Global Operations, United Kingdom Health Security Agency, London, UK; s Institute of Infection, Veterinary and Ecological Sciences, University of Liverpool, UK; t International Livestock Research Institute, Addis Ababa, Ethiopia

**Keywords:** National Bridging Workshop, One Health, Somalia, Health systems strengthening, Multisectoral coordination, International health regulation (IHR), Performance of veterinary services (PVS)

## Abstract

**Background:**

National Bridging Workshops (NBW) are a tool for reviewing collaboration gaps between line ministries relevant to the One Health framework.

**Methods:**

The NBW for Somalia was held on November 11–13, 2023 in Nairobi, Kenya with support from WHO and WOAH. Participants included representatives from the Somali government both national and sub-national (including Ministry of Health; Ministry of Livestock, Forestry and Range; Ministry of Agriculture and Irrigation; and Ministry of Environment and Climate Change). Other participants included representatives from non-governmental organizations, academia and the quadripartite. Structured sessions guided participants through a step-by-step process, starting from identifying gaps to collectively developing solutions. The design of these sessions aimed to foster active engagement and collaboration with the outcomes of each session contributing to the subsequent one.

**Results:**

A total of 60 participants partook in the exercise, representing human health (35%), animal health (27%), agriculture (13%), environmental health (7%) and other relevant sectors (18%). Eighty-three percent of participants represented the national level and 17% the sub-national level. The collaborative effort yielded a joint roadmap comprising 36 activities and 11 objectives. Priority objectives included: development of national joint surveillance systems for selected One Health threats (41/47 votes, or 87% of the total votes); establishment of a high-level ministerial system to govern and coordinate One Health activities (30/47; 64%); and establishment of emergency funding structures for priority zoonotic diseases along with development of a 5-year national investment plan for One Health (27/47; 57%). A total of 94% of activities required low or moderate cost to be implemented, and 90% of activities were identified to have a likely high impact on multisectoral collaboration. The timeline for implementing the activities is projected to span one to two years.

**Conclusion:**

The workshop promoted high-level engagement, national ownership, and leadership in addressing health threats at the human-animal-environment interface. The resulting co-created roadmap will be integrated into the National Action Plan for Health Security, supporting ongoing One Health efforts in Somalia.

## 1 Introduction

Somalia, like many low-and-middle-income countries (LMICs), faces heightened vulnerability to epidemic and pandemic risks, rooted in a complex interplay of factors entrenched within the One Health paradigm [1,2]. These include high reliance on animal husbandry, weak state capacity, fragile health systems and inadequate border control measures affecting human and livestock mobility [1]. In Somalia, this is further exacerbated by extreme weather events and climatic disasters such as droughts, floods and cyclones, coupled with recurrent epidemics owing to poor health system capacity for prediction, prevention, early detection and response [1]. Furthermore, the nation suffers from inadequate delivery of essential human and animal health service, evidenced by low immunization uptake for humans (e.g., measles, polio, cholera), intermittent livestock vaccination campaigns, and weak capacity to detect and respond to epidemic-prone diseases, particularly zoonoses [3,4].

The One Health paradigm underscores the interconnectedness of human, animal, and environmental health, and emphasizes the need to address these vulnerabilities holistically [5]. Despite increasing focus to strengthen One Health initiatives in Somalia, structural factors associated with individual, organizational and network issues have hampered the implementation and operationalization of One Health at federal, state and community levels [6]. These factors include, but are not limited to, lack of funding and political will, power dynamics, and organizational structures (system and policy based), along with the existing professional, sectoral and institutional silos and lack of community inclusion and participation [5,6]. Additionally, there is a lack of understanding of ecological processes that sustain pathogens in the environment, particularly at the interface between livestock and human populations [7].

Globally, various tools assess the capacities of human and animal health sectors to address health threats. The human health sector uses the International Health Regulations (IHR) Monitoring and Evaluation Framework (MEF) developed by the World Health Organization (WHO). This includes the IHR States Parties Self-Assessment Annual Report (SPAR) and the Joint External Evaluation (JEE) tools to assess core public health capacities [8,9]. Somalia has the lowest IHR core capacity and Global Health Security (GHS) indices globally, scoring 33 out of 100 and 16 out of 100, respectively [11]. In the 2016 JEE, 54% of indicators were rated as at “No Capacity” and 38% as “Limited Capacity”. In comparison, the animal health sector uses the Performance of Veterinary Services (PVS) Pathway developed by the World Organisation for Animal Health (WOAH; founded as OIE) [10]. Somalia recently jointly conducted the PVS evaluation along with the veterinary legislation support mission in October 2023.

Although these evaluation frameworks cover some aspects of One Health, specific tools are needed to assess and operationalize collaboration at the human-animal-environment interface for improved health security. National Bridging Workshops (NBW) provide a formal consultation and structured exercise bringing together national and sub-national stakeholders across key line ministries and other relevant sectors involved in One Health domains to improve cross-sectoral coordination and collaboration in the pursuit of improved national health security. This article describes how Somalia leveraged the previous JEE and PVS assessments to engage stakeholders in strengthening multisectoral collaboration through the NBW. The overall aim of the exercise was to strengthen multisectoral collaboration at the human-animal interface while improving the country's compliance to international standards and regulations. The specific aims are included in Supplementary Table 1.

## 2 Methods

### 2.1 Pre-workshop planning

In June 2023, the One Health National Level Technical Working Group (OHNLTWG), comprising relevant line ministries in the One Health domain and led by the Ministry of Health and Human Services (hereafter “Ministry of Health” or MoH) of the Federal Government of Somalia jointly requested the quadripartite (WHO, WOAH, Food and Agriculture Organization of the United Nations [FAO], and United Nations Environment Programme [UNEP]) through WHO to convene the NBW. This request was made with the endorsement and collaboration of other relevant ministries, reflecting the core principles of the One Health approach. Following this, a series of virtual meetings were organized with representatives from the quandripartite as well as the OHNLTWG. The latter comprises representatives from the Somalia's Federal ministries including MoH; Ministry of Livestock, Forestry and Range (MoLFR); Ministry of Environment and Climate Change (MoECC); and Ministry of Agriculture and Irrigation (MoAI). The main objectives of the pre-workshop meetings were to present the approach and process of the workshop to the country ministry representatives, validate the agenda, ensure a minimum number of required participants, and emphasize the national ownership and leadership of the overall process.

As part of pre-workshop planning, the OHNLTWG proposed and developed scenarios for use in the workshop. A consensus was reached among the organising team to include Rift Valley fever (RVF), anthrax, rabies, and antimicrobial resistance (AMR) as case studies. Rift Valley fever and anthrax were selected because they were ranked among the top diseases in the recent multisectoral zoonotic disease prioritization exercise for Somalia [1]. Although not ranked among top zoonoses in this exercise, rabies was included as a case study in the NBW because it is under surveillance and has caused outbreaks in the past. The selection of AMR was based on its intricate challenges at the convergence of human, animal, and environmental health. Consequently, controlling AMR requires comprehensive, collaborative, and effective responses across the interconnected health domains.

### 2.2 Participant selection

Participant selection was sector-specific: MoH selected participants from the human health sector; MoLFR selected those from the animal health sector; MoECC selected participants from the environmental sector; and MoAI selected participants from the agricultural sector. Through consensus, five out of six states of the country were selected to represent the subnational and local perspectives. The main selection criteria for state level participation were strong livestock activity (either for economic or domestic purposes), frequent human-animal interaction, and a record of at least one zoonotic disease outbreak in the past. Invitation letters for all participants were signed by the WHO country office, which received the participant list from the MoH through the Chair of the OHNLTWG (first author). For groupwork, participants were allocated into five subgroups with balanced representation across sectors and organizational levels.

### 2.3 Workshop process

The three-day workshop took place from November 11–15, 2023, in Nairobi, Kenya. The choice of Nairobi as the venue was made with the full agreement from the government of Somalia due to several reasons, including logistical proximity of WHO and WOAH offices, enhanced collaboration and safety of participants.

**Fig. 1:**
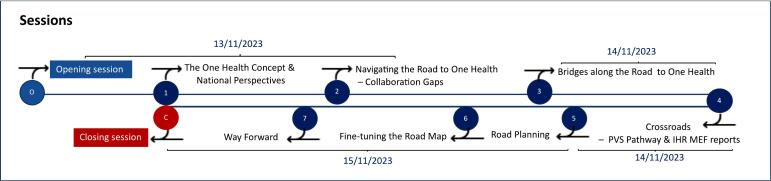
The timeline of the seven sessions of National Bridging Workshop for Somalia, held in Nairobi, Kenya, November 13–15, 2023.

The workshop was structured into seven sessions that were organized in a step-by-step process progressing from gap identification to the development and adoption of a joint roadmap for the enhancement of multisectoral collaboration to prevent and control zoonotic diseases (Fig. 1).

Session 1 provided an introductory overview to set the scene with a short presentation outlining the workshop's approach and methodology followed by videos presenting the concept and history of One Health. Subsequently, representatives from UNEP shed light on the environmental dimensions of One Health in the region. Moreover, insights were shared by national experts from Public Health Services, Veterinary Services, and Agricultural Services presenting their structure, priorities, and capacities.

In Session 2, participants were divided into four groups with mixed participants from each sector at different levels (central, provincial, district). Each group was provided with a case study developed in the planning phase. This session provided the opportunity to discuss the management of One Health threats at the human-animal-environment interface and identify strengths and weaknesses in the current collaboration among different sectors for key technical areas.

Session 3 started with videos presenting the IHR and related assessment tools (SPAR and JEE) as well as the PVS pathway (PVS Evaluation and PVS Gap Analysis). These were projected to the participants to help them reach the same level of understanding of the two frameworks. The differences and connections between these tools were explained via a comparative table accompanied by extracts of JEE and SPAR, highlighting their shared structure and process. A large matrix (IHR-PVS matrix), cross-connecting the indicators of the IHR MEF (in rows) and the indicators of the PVS Evaluation (in columns) was set-up and introduced to the participants. Using an interactive approach, working groups were asked to plot their technical area cards (from the session 2 exercise) into the matrix by matching them with their corresponding indicators. For the remaining sessions of the workshop, new working groups were formed, this time by technical area, to cover a maximum aspect of the collaboration that required improvement.

In Session 4, the newly formed groups extracted the main findings, gaps, and recommendations relevant to their technical area from the PVS Evaluation and JEE reports.

In Session 5, using all the information collected in sessions 2, 3 and 4, groups identified priority SMART (specific, measurable, achievable, realistic, and time-bound) objectives and joint activities that should be conducted to address the identified gaps and to improve multisectoral collaboration.

In Session 6, the groups engaged in a participatory process to fine-tune the objectives and activities outlined by all the groups through a World Café exercise.

Finally, in Session 7 a plenary was organized to discuss on the way forward, and to provide context on streamlining the joint roadmap developed into the existing national frameworks to support its implementation. This session was entirely facilitated by national stakeholders. Following the initial consolidation of the joint roadmap, a rapid prioritization exercise was then conducted based on cost-effectiveness and operational capacity and impact. Each participant selected five objectives considered to be of the highest priority, indicating their choices by placing stickers onto corresponding activity cards. The proportion of voters who prioritized each objective was then calculated as the number of votes for a specific objective divided by the total number of votes.

The workshop was facilitated by two lead facilitators (one from WHO Eastern Mediterranean Regional Office [EMRO] and one from WOAH Sub-Regional Representation for Eastern Africa) and three national facilitators (one from the MoH, one from the MoLFR, and one from MoAI). The comprehensive WHO-WOAH standardized toolkit, comprising posters, technical cards, fact sheets, stationery supplies, facilitator manuals, participant handbooks, and assessment reports (JEE and PVS), was extensively utilized throughout the workshop [12]. Throughout group discussions and plenary sessions, facilitators supervised groups without interfering, ensuring unbiased discussions and allowing participants to autonomously uncover locally grounded solutions. The purpose was to cultivate a conducive environment for participants to identify country specific and effective collaboration strategies tailored to Somalia's systems and context.

### 2.4 Evaluation

An evaluation of the workshop was conducted among participants to assess the workshop with the use of Google template. The questionnaire consisted of 9 questions covering various aspects such as satisfaction with the workshop content, structure/format, materials, methods, facilitation, and organization. Furthermore, it assessed the workshop's impact on technical skills and knowledge, its influence on participants' work, and overall collaboration within the country. Additionally, an open text section was included to gather suggestions for improvement.

### 2.5 Ethical considerations

The IHR-PVS workshop was determined to be non-research by National Research Ethical Review Board (NRERB), MoH of the Federal Government of Somalia and therefore exempt from IRB review. All methods were performed in accordance with the relevant guidelines and regulations or declaration of Helsinki [13].

## 3 Results

### 3.1 Participants

**Fig. 2:**
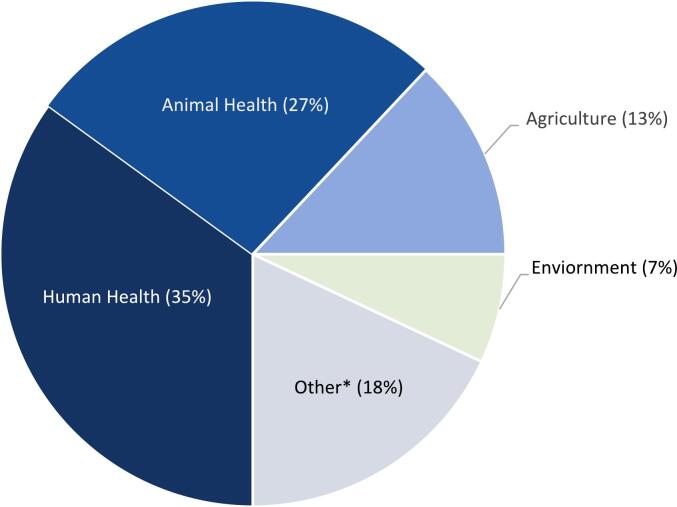
Stakeholder representation of the National Bridging Workshop for Somalia, held in Nairobi, Kenya, November 13–15, 2023. * Other includes policymakers, law enforcement, community leaders, budget planners, and other implementers.

The NBW workshop for Somalia had a total of 60 participants, 8 facilitators, and 3 observers. The organizational affiliations of participants are given in Table 1**.** As a proportion of participants, there was 35% representation from the human health sector, 27% from the animal health sector, 13% from the agriculture sector and 7% from the environmental health sector (Fig. 2). Additionally, participants from the Ministry of Interior Affairs and Reconciliation; academic and research institutions in Somalia (Somali National University, SIMAD University, Amoud University) and abroad (University of Liverpool and International Livestock Research Institute); and NGOs (Global Implementation Solutions and Vétérinaires Sans Frontières Suisse) participated in the workshop. National experts who have participated in PVS or JEE missions and technical partners such as WHO, WOAH, FAO and UNEP were also represented. Most participants (83%) were from the national level, with 20% from state level and 6% from international level.

**Table 1:** Participating organizations in the National Bridging Workshop for Somalia, held in Nairobi, Kenya, November 13–15, 2023. Note that some participants had dual affiliations. FAO, Food and Agriculture Organization; UNEP, United Nations Environmental Programme; WOAH, World Organization for Animal Health; WHO, World Health Organization.

Organization
**Participants**
***Government of Somalia***
*Federal government of Somalia*
Ministry of Health
National Institute of Health
Ministry of Livestock, Forestry and Range
Ministry of Environment and Climate Change
Ministry of Agriculture and Irrigation
Ministry of Internal Security
*Federal member state governments of Somalia*
Ministry of Health, Puntland State
Ministry of Health, Galmudug State
Ministry of Health, Banadir Administration
Ministry of Health, Somaliland
Ministry of Livestock, Forestry and Range, Jubaland State
Ministry of Livestock, Forestry and Range, Hirshabelle State
Ministry of Livestock, Forestry and Range, Southwest State
***Academic institutions from Somalia***
Somali National University, Somalia
SIMAD University, Somalia
Amoud University, Somaliland
**Facilitators**
Ministry of Health, Somalia
Ministry of Livestock, Forestry and Range, Somalia
Ministry of Environment and Climate Change, Somalia
Ministry of Agriculture and Irrigation, Somalia
WHO Eastern Mediterranean Regional Office (EMRO), Egypt
WHO Somalia Country Office
WHO Kenya Country Office
WOAH Sub-Regional Representation for Eastern Africa, Kenya
FAO Somalia Country Office
FAO Headquarters, Italy
Unlimit Health, UK
Royal Veterinary College, University of London, UK
**Advisors**
WOAH Sub-Regional Representation for Eastern Africa, Kenya
FAO Somalia Country Office FAO Headquarters, Italy
Global Implementation Solutions (GIS), Kenya
Vétérinaires Sans Frontières Suisse (VSF-Suisse), Kenya
UNEP Africa Office, Kenya
University of Liverpool, UK
International Livestock Research Institute (ILRI), Ethiopia

### 3.2 High-level engagement and country ownership

Opening remarks were delivered by key representatives from the relevant federal line ministries, WHO, and WOAH. Briefly, the chairperson (first author) highlighted the urgent necessity for collaborative efforts across various sectors to effectively address health threats at the animal-human-environment interface. Following this, an introductory session allowed participants to articulate commitments to improving collaboration and informing strategies for policymakers. In addition, the national taskforce also moderated the working groups alternating responsibilities between the three sectors on a daily basis. Furthermore, the concluding session focused the way forward, government ownership, and future implementation of the roadmap.

### 3.3 Assessing levels of collaboration

**Fig. 3:**
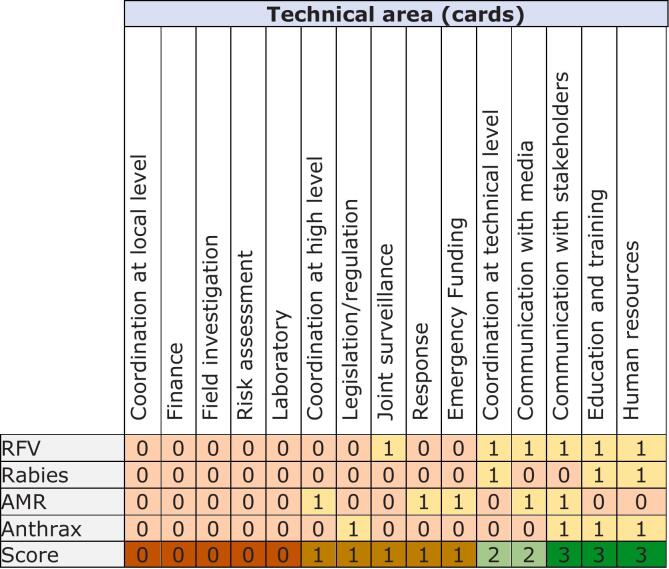
Levels of collaboration for 15 key technical areas as assessed for 4 case scenarios in the National Bridging Workshop for Somalia, held in Nairobi, Kenya, November 13–15, 2023. 0, collaboration needing improvement; 1, some collaboration; 2, average collaboration; 3, good collaboration.

During the second session, four groups were formed based on the developed case studies and participants were asked to self-assess their collaboration in 15 key technical areas. The findings of this activity are shown in Fig. 3. Collaboration scores were generally low across all technical areas for each case study.

### 3.4 Linking IHR and PVS sector-specific goals

In the third session, participants used the cards from the second session to plot collaboration levels into the IHR-PVS matrix by matching them with corresponding indicators. This enabled stakeholders to visualize matching areas between the two sectors and their respective frameworks. A plenary analysis of the outcome revealed clear clusters of gaps and highlighted that most gaps were not disease-specific but systemic.

A subsequent plenary analysis prioritized seven technical areas showing the most important gaps for the roadmap. These included: coordination at high, technical, and local levels; joint surveillance and laboratory; response and field investigation; and finance and emergency funding. In addition, human resources and education and training were included in each group as these aspects are cross-cutting issues. The session's outcome emphasized that the identified gaps were mainly systemic, rather than related to any specific disease.

In the fourth session, the human health sector participants consulted the PVS Evaluation report, and the animal health sector participants consulted the JEE report to extract key gaps and recommendations relevant to the development of the Joint One Health roadmap to support the operationalization of the One Health approach.

### 3.5 Outputs of the IHR-PVS NBW

A total of 11 objectives and 33 activities were developed for the roadmap (see Supplementary Table 2). Table 2 summarizes the total number of activities according to their level of cost and potential impact. A total of 31 (94%) activities required low or moderate cost to be implemented, and 30 (90%) activities were identified to have potential high impact on multisectoral collaboration.

**Table 2:** Number of activities in the road map by cost/difficulty in implementation and impact as identified in the National Bridging Workshop for Somalia, held in Nairobi, Kenya, November 13–15, 2023.

Level	Number of activities according to level of cost	Number of activities according to level of impact
Low	18	0
Moderate	10	3
High	5	30
Total	33	33

**Fig. 4:**
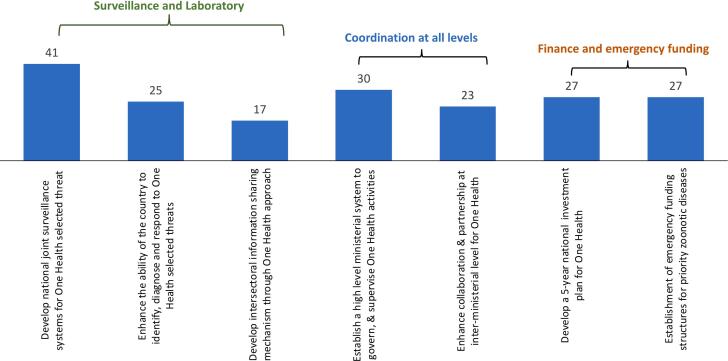
Priority objectives of the One Health roadmap, by technical areas, as identified in the National Bridging Workshop for Somalia, held in Nairobi, Kenya, November 13–15, 2023. Numbers indicate the number of people who identified that objective as a priority. A total of 47 participants contributed their votes.

**Fig. 5:**
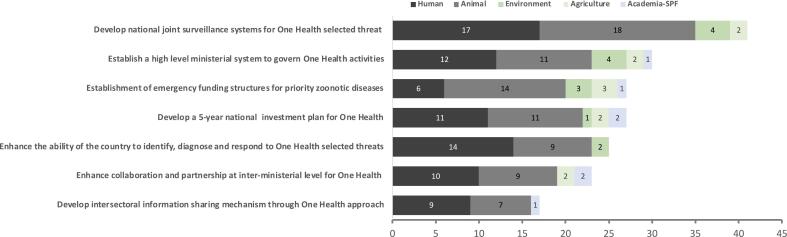
Priority objectives identified in the One Health roadmap, as prioritized by each sector in the National Bridging Workshop for Somalia, held in Nairobi, Kenya, November 13–15, 2023. Numbers indicate the number of people who identified that objective as a priority. SPF (Somali Police Force).

A total of 47 participants contributed their votes to the rapid prioritization exercise. The priority objective with the most votes (41/47; 87%) was the development of national joint surveillance systems for selected One Health threats (Fig. 4). The second objective was related to the establishment of a high-level ministerial system to govern and supervise One Health activities which received 30 votes (64% of the total votes). Lastly, 27 participants (57%) selected the development of a 5-year national investment plan for One Health and establishment of emergency funding structures for priority zoonotic diseases. The breakdown of the results of the prioritization vote by sector is given in Fig. 5.

### 3.6 Workshop evaluation

Evaluation data revealed an overwhelmingly positive response (see Supplementary Figure 1): 96% of participants reported being “satisfied” or “fully satisfied” with the workshop, while 94% anticipated “significant impact,” and 98% expected “very high impact” on their respective departmental work. Furthermore, 99% of participants acknowledged that the workshop improved their understanding of the One Health approach. Additionally, high levels of support (95%) were expressed for the integration of other sectors including the Ministry of Internal Security and the Ministry of Fisheries and Marine Resources into the current OHNLTWG. Finally, the workshop proved instrumental in augmenting participants' comprehension of key frameworks, with 98% and 85% reporting improved understanding of the IHR 2005 and the PVS assessment tools, respectively.

## 4 Discussion

The successful convening of the NBW for Somalia in November 2023, at the request of the OHNLTWG led by the MoH of the Federal Government of Somalia, reflects high-level engagement and government ownership in addressing health threats at the human-animal-environment interface. This collaborative effort, facilitated by the quadripartite, signifies the importance of a multi-sectoral approach to global health security and operationalization of the One Health approach [5]. The fact that Somalia requested external financial support from the quadripartite to conduct the NBW reflects the chronic underfunding of pandemic preparedness specifically and low government spending on health more generally which remains the lowest globally ($1 per person) [14]. However, it is worth noting that most NBW in Africa are dependent on donor funding as are most One Health initiatives on the continent [15].

The involvement of the OHNLTWG with representatives from all relevant line ministries along with comprehensive preparatory sessions reinforced the principles of national ownership, government buy-in and leadership. This proactive involvement served as a pivotal mechanism in anchoring the joint roadmap into an existing framework, thereby enhancing its uptake and ensuring a wider integration into broader health strategies. Similar observations have been reported in several countries including Kazakhstan and Nigeria where the collaborative initiatives identified during the NBW were integrated into their respective One Health Strategic Plans. Similarly, in Jordan and Pakistan, the roadmap activities outlined during the NBW were effectively incorporated into the National Action Plan for Health Security (NAPHS) [12].

Such strategic alignment highlights the practical utility and relevance of workshop outcomes in shaping and reinforcing national health agendas. However, such integration was not feasible in Somalia as both One Health Strategic planning and NAPHS, a product of the SPAR/JEE process, predated the current NBW. Nonetheless, it was recommended that the activities from the joint roadmap be incorporated as complementary activities in the annual operational plans. In any case, the NBW provided sectors the opportunity to review and identify critical gaps in coordination mechanisms among relevant ministries, agencies, and stakeholders to address zoonotic diseases. Results from several NBWs indicated the need for strong and sustainable multisectoral coordination mechanisms to tackle future outbreaks of zoonotic origin. To enhance coordination, the WHO-FAO-WOAH tripartite developed the Multisectoral Coordination Mechanism Operational Tool (MCM-OT), piloted in several countries including Kenya, the Gambia, Kazakhstan and Armenia [16]. The Somali government should utilize this tool to evaluate and strengthen its coordination mechanism to support One Health activities.=+

The success of the NBW in Somalia was due to the selection and convening of a diverse set of stakeholders and subject matter experts as outlined by the workshop guidelines. This inclusive approach extended beyond governmental bodies to include academic institutions, local and international NGOs, the quadripartite organizations, and community representatives, enriching the discussions. Although the workshop achieved a balanced representation across human and animal health sectors, a notable disparity was observed in the representation from the environmental health sector. This departure from the recommended distribution underscores the need for a more inclusive approach to ensure proportional representation from all relevant sectors [12]. The Federal Member State representations from Puntland, Galmudug, Somaliland, Hirshabelle, Jubbaland, and Banadir administration ensured the workshop captured some sub-national level viewpoints. However, sub-national representatives made up a relatively low proportion of workshop participants (17%). Future activities should strive to ensure more balanced representation of sub-national stakeholders given their importance as implementers of One Health. The involvement of national experts with experience in JEE and PVS, alongside the quadripartite provided invaluable insights and expertise. Furthermore, the sector-specific selection process, with ministries responsible for selecting their own representatives facilitated targeted and focused discussions on key health security issues. Overall, the NBWs success in Somalia can be attributed to its inclusivity, sector-specific dialogue, and comprehensive subnational representation, all of which collectively enhanced its effectiveness in addressing complex national health security challenges in the country.

The need for regular interaction between human health, animal health, and environmental sectors has been widely recognised and systematically recorded in JEE and PVS recommendations [17,18]. By utilizing user-friendly materials including visual cards, matrixes, participant support materials such as the participants handbook and the interactive nature of the workshop and structured group sessions, impediments were dismantled, enabling transparent dialogue and inclusive participation thereby promoting interdisciplinary problem-solving. This approach facilitated brainstorming and the identification of practical, contextually relevant solutions. Overall, the workshop exemplified an effective model for promoting intersectoral collaboration and generating sustainable interventions.

The projection of videos illustrating the IHR alongside associated assessment tools such as SPAR and JEE, as well as PVS pathway including PVS Evaluation and PVS Gap Analysis, improved same level of understanding of these frameworks among all participants. Subsequent clarification of the distinctions and synergies among these tools via a comparative table cross-referencing indicators from the IHR MEF with those from the PVS Evaluation thereby facilitated the identification of bridges between these frameworks. Similar results were noted in several NBW workshops conducted across Africa and elsewhere in Asia, where the IHR-PVS matrix enabled stakeholders to easily visualize the links between the two sectors and the two frameworks [12].

Furthermore, the consultation of each sector with their specific JEE or PVS Evaluation reports facilitated the extraction of pertinent gaps and recommendations for the joint roadmap conducive to operationalizing the One Health approach. The IHR-PVS matrix also allowed participants to highlight complementarities and identify synergies between the two frameworks; increased awareness of the IHR (2005) and PVS across all sectors and improved the understanding of Veterinary Services in the implementation of IHR. This practice facilitated the integration of the NBW outcomes enabling necessary adjustments at the human-animal-environment interface [17]. Similar observations were documented in Cameroon's NBW [15].

Our study limitations included a limited timeframe and insufficient representation of the environmental sector and sub-national government, which is critical for navigating complex issues and implementing One Health initiatives effectively. Moreover, the reliance on external funding sources may impede sustainability, necessitating diverse financial streams and long-term planning to ensure sustainability. The generalizability of workshop findings beyond Somalia's context is demarcated by regional dynamics, political landscapes, and resource disparities in other settings. Recognizing these contextual nuances is pivotal for tailoring One Health strategies to specific environments and optimizing their impact. Mitigating these constraints demands heightened stakeholder engagement, diversified funding channels, methodological refinements, and longitudinal evaluation strategies. These measures are essential for the efficacy and sustainability to enhance One Health implementation and the contribution to the global health security capacities.

## 5 Conclusion

The NBW for Somalia highlighted the importance of the One Health approach to effectively tackle threats at the human-animal-environment interface. The workshop was an opportunity to identify collaboration challenges. The diverse participation from governmental bodies, academia, NGOs, and international organizations enriched the discussions, leading to the development of a joint roadmap aiming at strengthening collaboration with the use of the One Health approach. However, challenges such as the underrepresentation of certain groups and reliance on donor funding were noted, underscoring the need for a more inclusive approach and diversified funding sources for sustainable One Health initiative in Somalia.

## Funding

The National Bridging Workshop for Somalia was funded by the 10.13039/100004423World Health Organization (WHO), the World Organization for Animal Health (WOAH), and the Food and Agriculture Organization (FAO).

## CRediT authorship contribution statement

**Abdinasir Yusuf Osman:** Writing – original draft, Data curation, Conceptualization. **Asma Saidouni:** Writing – review & editing, Data curation. **Lillian Wayua Wambua:** Writing – review & editing, Formal analysis. **Heba Mahrous:** Writing – review & editing. **Sk Md Mamunur Rahman Malik:** Writing – review & editing. **Mutaawe Lubogo:** Writing – review & editing. **Reinhilde Van de Weerdt:** Writing – review & editing. **Ali Hadji Adam:** Writing – review & editing. **Hassan Hussien Mohamed:** Writing – review & editing. **Khadija Al Makhzoumi:** Writing – review & editing. **Guled Abdijalil Ali:** Writing – review & editing. **Mohamed Omar Nur:** Writing – review & editing. **Sonia Fevre:** Writing – review & editing. **Gerald Mucheru:** Writing – review & editing. **Sophycate Njue:** Writing – review & editing. **Alain Okito Mosindo:** Writing – review & editing. **Kaitlin Sandhaus:** Writing – review & editing. **Rosita Claesson Wigand:** Writing – review & editing. **Claire Standley:** Writing – review & editing. **Erin Sorrell:** Writing – review & editing. **Richard Kock:** Writing – review & editing. **Javier Guitian:** Writing – review & editing. **Alimuddin Zumla:** Writing – review & editing. **Osman Dar:** Writing – review & editing. **Siobhan M. Mor:** Writing – review & editing.

## Declaration of competing interest

The authors declare the following financial interests/personal relationships which may be considered as potential competing interests.

AHA is the Minister of Health of Somalia; HHM is the Minister of Livestock, Forestry and Range of Somalia; KM is the Minister of Environment and Climate Change of Somalia; GAA is the Director General of the Ministry of Health of Somalia; MON is the Director General of the Ministry of Livestock, Forestry and Range of Somalia; AYO is the Chair of the One Health National Level Technical Working Group (OHNLTWG) of Somalia.

SMM is on the editorial board for the journal, One Health.

## Data Availability

Data will be made available on request.

## Appendix A Supplementary data

Supplementary materials accompanying the manuscript on the IHR-PVS National Bridging Workshop for Somalia. Image 1
