# Supplementary material for: IHR-PVS National Bridging Workshop for Somalia: An interactive and participatory approach for operationalizing the One Health roadmap
Source: One Health. 2024 Jul 14;19:100858. doi: 10.1016/j.onehlt.2024.100858 (PMC11327579; doi:10.1016/j.onehlt.2024.100858)
Supplement: Supplementary file 1 — Supplementary materials accompanying the manuscript on the IHR-PVS National Bridging Workshop for Somalia. [file mmc1.docx]

Supplementary Table 1: Specific aims of the NBW for Somalia

| 1. | To identify and review the current collaboration gaps of the federal line ministries involved in One Health in key technical areas. |
| --- | --- |
| 2. | To develop a joint roadmap of corrective measures. |
| 3. | To develop a strategic investment plan to improve the collaborative work at the animal-human-environment interface. |
| 4. | To improve One Health approach understanding and the need for multisectoral collaboration at the animal-human-environment health interface. |
| 5. | To promote dialogue, coordination, and collaboration, recognising the crucial need for the animal and human health sectors to come together to strategically identify areas for joint actions and develop a synergistic approach. |
| 6. | To strengthen the inter-sectoral collaboration through improved understanding of respective roles and mandates. |
| 7. | To enhance strategic planning and investments (including the National Action Plan for Health Security) based on the structured and agreed identification of needs and options for improvement. |

Supplementary Table 2: NBW roadmap for Somalia - objectives and actions identified per technical areas

| **Activity** | **Timeline** | **Difficulty** | **Impact** | **Responsibility** | **Process** |
| --- | --- | --- | --- | --- | --- |
| **coordination at high, technical and local level** | | | | | |
| **Objective 1: To establish a High level Ministerial (MoH, MoLFR, MoECC and MoAI) system to govern, manage, coordinate and supervise one health activities** | | | | | |
| **Establish a High level Ministerial One Health steering committee** | Q1 2024 | **+** | **+++** | MoH, MoLFR, MoECC, and MoAI | - Conduct consultation meeting to agree on establishment of a high level ministerial steering committee - Develop ToRs for the steering committee |
| **Establish a National One Health secretariat** | Q1 2024 | **+** | **+++** | MoH, MoLFR, MoECC, and MoAI | - Nominate focal points from each sector to establish a national one health secretariat - Develop the ToR for national one health secretariat - Organize Multi sectoral Coordination Mechanism (MCM) workshops |
| **Develop a Memorandum of Understanding on mutual collaboration** | Q1-Q2 2024 | **+** | **+++** | Legal and Technical Departments of MoH, MoLFR, MoECC, and MoAI | - Agree to draft MoU at high level - Draft MoU including Animal and Human Health, plant, and environment inputs - Conduct internal consultations within each sector - Approve the MoU by all involved parties - Commence MoU |
| **Objective 2: To enhance collaboration and partnership at inter-ministerial level for implementation of one health activities** | | | | | |
| **Establish multi-sectorial technical working groups for zoonotic diseases, AMR, Food safety involving all the stakeholders at federal level** | Q3 2024 | **+** | **+++** | MoH, MoLFR, MoECC, and MoAI | - Nominate focal points from each stakeholder to join the different technical working groups. - Develop the membership criteria to join the different committees - Invite international, regional partners, academia, private sector, and communities |
| **Establish multi-sectorial technical working groups for zoonotic diseases, AMR, Food safety involving all the stakeholders at state level** | Q1 2025 | **++** | **++** | MoH, MoLFR, MoECC, and MoAI | - Nominate focal points from each stakeholder to join the different technical working groups. |
| **Review and further develop strategies and plans for one health technical areas and align with existing national plans such as the national Action Plan for Health Security (NAPHS)** | Q3 2024 | **+** | **+++** | MoH, MoLFR, MoECC, and MoAI | - Desk review the existing strategies and plans (e.g. NAPHS, AMR NAP and Sectorial strategic plans) |

| **FIELD INVESTIGATION AND RESPONSE** | | | | | |
| --- | --- | --- | --- | --- | --- |
| **Objective 3: Establishing framework for fields investigation and response** | | | | | |
| **Develop joint contingency plans for priority zoonotic diseases** | Q2 2024 | **+++** | **+++** | OH TWG, Animal health sector, Human health sector, Environmental sector, Agricultural sector, SODMA Academia, Community members, International Agencies | - Develop contingency plan/s involving all concerned parties with different scenarios, with the collaboration of all stakeholders including NGOs, and international organizations (WHO, WOAH, FAO…) - Validate the contingency plan - Dissemination of contingency plan to all involved parties |
| **Conduct Joint Risk Assessment for the priority zoonotic disease** | Q4 2024 | **+++** | **+++** | OH TWG, Animal health sector, Human health sector, Environmental sector, Agricultural sector, SODMA Academia, Community members, International Agencies | - Conduct a ToT training (25 trainees) at National level on Joint Risk Assessment - Conduct 6 sub-national Joint Risk Assessment training for priority zoonotic and food-borne diseases |
| **Establish multi-sectoral Rapid Response Teams (RRTs) at national level and subnational level** | Q2 2024 | **+** | **+++** | OH TWG, Animal health sector, Human health sector, Environmental sector, Agricultural sector, SODMA Academia, Community members, International Agencies | - Activate the multisectoral RRT at national level. - Specify and identify the role and responsibilities - Establish a list of persons involved in RRT unit and a Roster list - Train the RRT on various One Health Approach and joint activities |
| **Map out a comprehensive assessment of available emergency response resources at the national to local levels** | Q2 2024 | **+** | **+++** | OH TWG, Animal health sector, Human health sector, Environmental sector, Agricultural sector, SODMA Academia, Community members, International Agencies | - Stakeholder engagement and identification of resources - Review of available resources |
| **Conduct training in line with the one health approach for national and sub-national RRTs** | Q4 2024 | **++** | **+++** | OH TWG, Animal health sector, Human health sector, Environmental sector, Agricultural sector, SODMA Academia, Community members, International Agencies | - Establish training plan - Selection of the expert (national & sub national experts) that are qualified in the field needed - Prepare training material by the experts - Conduct TOT training - Conduct the cascade training - Establish roster of trained individuals (database) |
| **Conduct a training on  One Health joint outbreak investigation** | Q4 2024 | **+** | **+++** | OH TWG, Animal health sector, Human health sector, Environmental sector, Agricultural sector, SODMA Academia, Community members, International Agencies | - Review existing training plan available of joint outbreak investigation - Develop/adapt a training plan for One Health workforce - Identify and engage trainers - Conduct a training at national and subnational levels. |
| **Integrate one health approach and FETP** | Q2 2024 | **++** | **+++** | OH TWG, Animal health sector, Human health sector, Environmental sector, Agricultural sector, SODMA Academia, Community members, International Agencies | - Map out the existing OH workforce development - Select the suitable curriculum FOR oh approach - Conduct TOT - Cascade the training |
| **Objective 4: Assess preparedness and response capacities for health threats at the human-animal-environment interface** | | | | | |
| **Conduct a national and subnational One Health tabletop simulation exercise on Public Health Emergency Operations Center (PHEOC) SOPs.** | Q4 2024 | **+** | **+++** | OH TWG, Animal health sector, Human health sector, Environmental sector, Agricultural sector, SODMA Academia, Community members, International Agencies | - Establish a plan of SimEx - Conduct SimEx, at national (24 participants) and 6 at subnational level - Evaluate the Simex |
| **Conduct a national and subnational One Health tabletop simulation exercise on contingency plans.** | Q4 2024 | **+** | **+++** | OH TWG, Animal health sector, Human health sector, Environmental sector, Agricultural sector, SODMA Academia, Community members, International Agencies | - Establish a plan of SimEx - Conduct 7 SimEx, 1 at national (24 participants) and 6 at subnational level - Evaluate the Simex |
| **Conduct joint Simex focusing on OH outbreak response** | Q2 2025 | **++** | **+++** | OH TWG, Animal health sector, Human health sector, Environmental sector, Agricultural sector, SODMA Academia, Community members, International Agencies | - Select the type of Simex - Identify participants - Implement the Simex - Evaluate the Simex |
| **Conduct an after-action review for zoonotic disease** | Q2 2025 | **+** | **++** | OH TWG, Animal health sector, Human health sector, Environmental sector, Agricultural sector, SODMA Academia, Community members, International Agencies | - Select the zoonotic priority disease outbreaks - Conduct after action review |
| **Conduct a national and subnational One Health tabletop simulation exercise on Public Health Emergency Operations Center (PHEOC) SOPs.** | Q4 2024 | **+** | **+++** | OH TWG, Animal health sector, Human health sector, Environmental sector, Agricultural sector, SODMA Academia, Community members, International Agencies | - Establish a plan of SimEx - Conduct 7 SimEx, 1 at national (24 participants) and 6 at subnational level - Evaluate the Simex |
| **Conduct a national and subnational One Health tabletop simulation exercise on contingency plans.** | Q4 2024 | **+** | **+++** | OH TWG, Animal health sector, Human health sector, Environmental sector, Agricultural sector, SODMA Academia, Community members, International Agencies | - Establish a plan of SimEx - Conduct SimEx, at national and subnational level |

| **SURVEILLANCE & LABORATORY** | | | | | |
| --- | --- | --- | --- | --- | --- |
| **Objective 5: Develop national joint surveillance System for one health related threats (Human, Animal, and Environment)** | | | | | |
| **Map the existing surveillance systems in different sectors (Zoonotic, food-borne diseases and AMR).** | Q1 2024 | **++** | **+++** | Directors of Health Departments in the relevant ministries | - Develop a checklist for mapping evaluation. - Visit the sectors (Health, Livestock, Agriculture and Environment) - Assess the existing surveillance system. - Conduct a workshop for harmonization and validate. |
| **Assign surveillance focal points for one health approach at national level and state level** | Q1 2024 | **+** | **+++** | Directors of Health Departments in the relevant ministries | - Directors will plan the process |
| **Develop national strategic plan for surveillance to the prioritized six zoonotic diseases.** | Q2 2024 | **+++** | **+++** | Directors of Health Departments in the relevant ministries | - Compile the previous strategic plan and upgrade the new one health surveillance strategic plan. - Recruit a consultant. - Conduct a workshop to develop the components. - Conduct a workshop for validation |
| **Objective 6: Enhance the country capacities to identify, diagnose, and detect One Health-related threats** | | | | | |
| **Upgrade laboratory facilities and equipment to meet international standards.** | Q2 2024 | **+++** | **+++** | Lab Directors | - Conduct mapping assessment to know the existing capacities (infrastructure, Equipment, Reagents and Human resources) for both animal and public health - Mobilize the resource to fill the gaps. - Implement quality assurance and quality control measures to ensure the accuracy of diagnostic results. - Create interlinking laboratory networking system locally and internationally. |
| **Establish or expand access to diagnostic testing for One Health-related threats.** | Q2-Q3 2024 | **++** | **+++** | Directors of National labs | - Supply reagents and equipment - Train the staff - Link the existing labs (Public and Private) with the one health system |
| **Assign lab focal point for one health approach, National and State** | Q1 2024 | **+** | **++** | National One health Focal Point and Directors of National labs | - One health Focal Point will nominate the lab focal points. |
| **Train laboratory technicians and professionals in One Health diagnostics and surveillance.** | Q2 2024 | **+** | **+++** | Directors of National labs | - Develop SOPs and Guidelines - Develop training plan - Seek the training budget |
| **Objective 8: Develop intersectoral information sharing mechanisms through one health approach.** | | | | | |
| **Link all current data based to IDSR for one health system for real time data sharing and analysis platforms** | Q4 2024 | **++** | **+++** | Head of Surveillance Unit in different ministries | - Assessment in all surveillance data platforms in different sectors like human health, animal health etc. - Create a joint platform to share data of all zoonotic diseases. - Develop Data Management and Analysis Systems: Implement a robust data management system to store, organize, and analyze the collected data. Develop data analysis tools and techniques to identify patterns, trends, and potential risks associated with one health threats. |
| **Establish Surveillance Networks: Create a network of surveillance sites across the country, including hospitals, clinics, veterinary clinics, farms, wildlife sanctuaries, and environmental monitoring stations** | Q1 2024 | **+** | **+++** | Different line ministries, private and public hospitals and civil society. | - Conduct a risk assessment. - Create a joint technical working group for surveillance network. - Head of Surveillance Units in different sectors should collaborate. |

| **FINANCE AND EMERGENCY FUNDING** | | | | | |
| --- | --- | --- | --- | --- | --- |
| **Objective 9: Develop a 5-year national investment plan for One Health** | | | | | |
| **Identify and map existing funding sources as well as priority one health areas with gaps for additional funding.** | Q1 2024 | **++** | **+++** | One health TWG and relevant stakeholder | - Development of a structured questionnaire to all relevant stakeholders. - Compile report of the findings. - Conduct a validation workshop involving high-level authority. - Mapping relevant stakeholders for funding |
| **Develop and endorse the draft 5-year investment plan for one health** | Q1 2024/  Q2 2025 | **++** | **+++** |  | - Hire consultant to develop a draft investment plan including emergency funding for zoonotic diseases. - Drafted plan to be presented to TWG and relevant stakeholders in a workshop. - Revisions adopted and incorporated into final draft. - Side meeting for investment partners to present roadmaps and contingency plans to partners and high-level decision makers. - Workshop to validate and endorse final version of investment plan. |
| **Objective 10: Decentralization of funding for one health Risk Communication and Community Engagement (RCCE) at district and regional levels** | | | | | |
| **Putting in place financial structures (MoU between National and State levels) to decentralize one health budget from national to all levels.** | Q4 2024 | **+++** | **+++** | Legal and Technical Departments of MHSP, MARD, and Ministry of Environment | - Draft MoU between National and State levels for the 4-line ministries. - Set up a working group including representatives from MoF and the parliament. - Conduct consultations in each sector. - Approve the MoU by all involved parties - Commence MoU |
| **Capacity building for staff working at regional level on autonomy for financial and HR management.** |  |  |  |  |  |
| **Objective 11: Establishment of emergency funding structures for priority zoonotic diseases** | | | | | |
| **Mobilize funds and resources for emergency contingency plans developed for priority zoonotic diseases.** | Q4 2024 | **+** | **++** | TWG from 4 core ministries and supporting ministries such as MoF, MoP | - Identification of partners to fund. |
| **Resource mobilization for operationalization of PHEOC** | Q4 2024 | **++** | **+++** | TWG and PHEOC team | - Draft concept notes clarifying key areas for funding. - Engage stakeholders and partners. |

***Difficulty of implementation:*** *Low +, Moderate ++, Very difficult +++* ***Impact:*** *Low impact +, Moderate impact ++, High impact +++*

Supplementary Figure 1: Evaluation of the NBW for Somalia, held in Nairobi, Kenya, November 13–15, 2023.
